# Postfracture survival in a population-based study of adults aged ≥66 yr: a call to action at hospital discharge

**DOI:** 10.1093/jbmrpl/ziae002

**Published:** 2024-04-09

**Authors:** Geneviève Vincent, Jonathan D Adachi, Emil Schemitsch, Jean-Eric Tarride, Nathan Ho, Rajvi J Wani, Jacques P Brown

**Affiliations:** Medical Affairs Division, Amgen Canada Inc., Mississauga, ON L5N 0A4, Canada; Department of Medicine, McMaster University, Hamilton, ON L8S 4L8, Canada; Division of Orthopaedic Surgery, Western University, London, ON N6A 3K7, Canada; McMaster Chair in Health Technology Management, Department of Health Research Methods, Evidence and Impact (HEI), McMaster University, Hamilton, ON L8S 4L8, Canada; Center for Health Economics and Policy Analysis (CHEPA), McMaster University, Hamilton, ON L8S 4L8, Canada; Programs for Assessment of Technology in Health (PATH), The Research Institute of St. Joe's Hamilton, St. Joseph's Healthcare Hamilton, Hamilton, ON L8N 4A6, Canada; Medical Affairs Division, Amgen Canada Inc., Mississauga, ON L5N 0A4, Canada; Research Division, Amgen Canada Inc., Mississauga, ON L5N 0A4, Canada; CHU de Québec Research Centre and Laval University, 2705 Boulevard Laurier, TR-83, Québec, QC L5N 0A4, Canada

**Keywords:** fracture prevention, osteoporosis, general population studies, screening, aging

## Abstract

Postfracture survival rates provide prognostic information but are rarely reported along with other mortality outcomes in adults aged ≥50 yr. The timing of survival change following a fracture also needs to be further elucidated. This population-based, matched-cohort, retrospective database study examined 98 474 patients (73% women) aged ≥66 yr with an index fracture occurring at an osteoporotic site (hip, clinical vertebral, proximal non-hip non-vertebral [pNHNV], and distal non-hip non-vertebral [dNHNV]) from 2011 to 2015, who were matched (1:1) to nonfracture individuals based on sex, age, and comorbidities. All-cause 1- and 5-yr overall survival and relative survival ratios (RSRs) were assessed, and time trends in survival changes were characterized starting immediately after a fracture. In both sexes, overall survival was markedly decreased over 6 yr of follow-up after hip, vertebral, and pNHNV fractures, and as expected, worse survival rates were observed in older patients and males. The lowest 5-yr RSRs were observed after hip fractures in males (66–85 yr, 51.9%–63.9%; ≥86 yr, 34.5%), followed by vertebral fractures in males (66–85 yr, 53.2%–69.4%; ≥86 yr, 35.5%), and hip fractures in females (66–85 yr, 69.8%–79.0%; ≥86 yr, 52.8%). Although RSRs did not decrease as markedly after dNHNV fractures in younger patients, relatively low 5-yr RSRs were observed in females (75.9%) and males (69.5%) aged ≥86 yr. The greatest reduction in survival occurred within the initial month after hip, vertebral, and pNHNV fractures, indicating a high relative impact of short-term factors, with survival-reduction effects persisting over time. Therefore, the most critical period for implementing interventions aimed at improving post-fracture prognosis appears to be immediately after a fracture; however, considering the immediate need for introducing such interventions, primary fracture prevention is also crucial to prevent the occurrence of the initial fracture in high-risk patients.

## Graphical Abstract

**Graphical Abstract:**
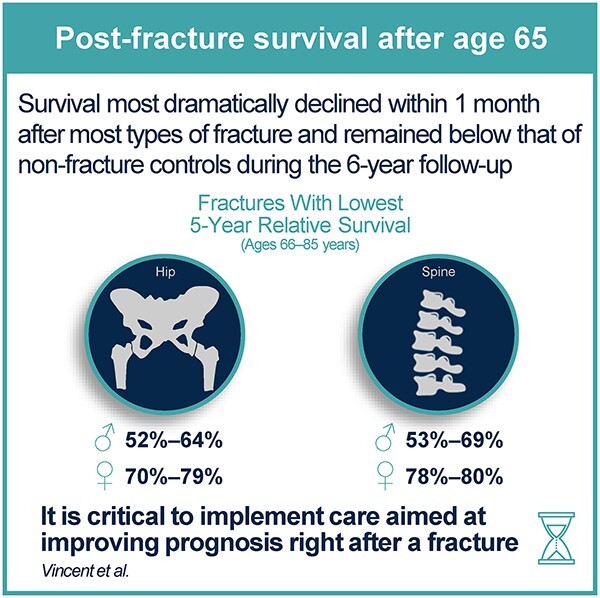

## Introduction

Fractures occurring at sites typically associated with osteoporosis in adults aged ≥50 yr significantly increase mortality.^1-14^ Initially, hip and vertebral fractures, and more recently proximal non-hip non-vertebral fractures (pNHNV)^12-15^ were found to increase mortality to an extent similar to that observed with other serious disease events such as myocardial infarction.^1-14^ Distal non-hip non-vertebral fractures (dNHNV), although a significant clinical event in this population owing to being a risk factor for subsequent fracture(s), were observed to increase mortality in a less-consistent fashion than other fracture types.^1^^,^^2^^,^^5^^,^^14^ However, although a robust body of evidence has accumulated about postfracture mortality outcomes in adults aged ≥50 yr, survival rates have rarely been reported.^6-9^ One- and 5-yr relative survival ratios (RSRs) are commonly reported in other disease areas, along with other mortality outcomes, because they provide unique information related to prognosis in patients after a disease event or diagnosis compared to similar people without the disease event or diagnosis.^16^^,^^17^ As such, RSRs are typically used in patient-provider conversations to discuss expected prognosis and appropriate interventions aiming to improve it and are monitored by public health agencies to identify improvements in disease management over time, such as use of more effective treatments in curbing disease progression.^16^^,^^17^

The aim of the current real-world population-based study was to characterize postfracture prognosis in adults aged ≥66 yr by examining 1- and 5-yr overall survival and RSRs separately for females and males, as well as for different age groups and fracture-site categories. Time trends in survival changes were also described, starting immediately after a fracture and up to 6 yr thereafter, to identify time periods critical for clinical interventions aiming to improve postfracture prognosis.

## Materials and methods

This descriptive observational study is based on previously described fracture and nonfracture cohorts.^3^^,^^18-20^ Its reporting was guided by the Reporting of Studies Conducted Using Observational Routinely Collected Health Data (or RECORD) statement.^21^^,^^22^

### Study design and data sources

This population-based, matched-cohort, retrospective database study was conducted in Ontario, Canada. The following administrative databases of Ontario’s public health care system were utilized to identify and describe fracture and nonfracture cohorts: Registered Persons Database, Discharge Abstract Database/Same Day Surgery, National Ambulatory Care Reporting System, and Ontario Health Insurance Plan. The Institute for Clinical Evaluative Sciences (ICES) Data Repository was used, which records public health care encounters in multiple record-level administrative datasets and links the datasets with an encrypted patient-specific identifier (ICES-specific key number). The ICES Data Repository encompasses publicly funded health services records for the population of Ontario, including its long-term care residents.^23^

### Participants

The fracture cohort included Ontario residents aged ≥66 yr who sustained an index fracture between January 1, 2011, and March 31, 2015. A minimum age cutoff was 66 yr because an initial study of this cohort examined osteoporosis treatment rates after and 1 yr prior to index fracture, which was based on public drug coverage in Ontario starting at the age of 65 yr^20^; a maximum predefined age cutoff was 105 yr. Fractures were identified from emergency or ambulatory care and acute hospital admissions, using International Classification of Diseases, 10th Revision (ICD-10) diagnostic codes for fracture as a main or admitting diagnosis or, as the main symptom, abnormal finding, or problem, if no definite diagnosis was made. Because the target patient population of the original cohort examined here^3^^,^^18-20^ comprised adults at risk of subsequent fracture, eligible patients included those who sustained an index fracture at a fracture site typically associated with osteoporosis. For the purposes of this study, the fracture cohort was divided into 4 fracture-site categories: (1) hip, (2) clinical vertebral, (3) pNHNV (pelvis, femur, sternum/rib/clavicle, and humerus/shoulder), and (4) dNHNV (tibia/fibula/knee, radius/ulna, and wrist) (Supplementary Methods).

Patients were excluded from the fracture cohort if they experienced multiple fractures during the same incident (ie, they could not be categorized in 1 of the above 4 fracture-site categories based on a single fracture site); experienced a fracture coded as pathologic or periprosthetic (Supplementary Methods) or high-trauma (high-trauma fractures were traditionally not considered to be associated with increased risk of subsequent fracture, only more recently^24^); experienced a fracture within 5 yr prior to their index event (to minimize the confounding effect of previous fracture on mortality outcomes); sustained a fracture of a small bone, including the skull, face, hand, or foot (small bone fractures are not known to be associated with increased fracture risk); were non-Ontario residents; had death date recorded prior to the index fracture date (ie, administrative error); or could not be matched with a nonfracture individual.

A nonfracture cohort was also assessed to calculate fracture-related RSRs. Each patient with an index fracture was matched 1:1 to an individual who did not sustain a fracture during the indexing period (January 1, 2011 to March 31, 2015) and within 5 yr prior to their index date. Exact matching (ie, not propensity-score matching) was performed based on the following prespecified variables: sex; age group (66–70, 71–75, 76–80, 81–85, and ≥ 86 yr); rural/urban residence; and comorbidities associated with fracture risk (ie, history of asthma, chronic obstructive pulmonary disorder [COPD], osteoarthritis, rheumatoid arthritis, psoriasis, spondyloarthritis, CKD, diabetes, myocardial infarction, stroke, and dementia at any time prior to index date; and history of cancer within 5 yr before index date), some of which are also typically associated with high mortality (eg, cancer, myocardial infarction, CKD, COPD, stroke, and diabetes). A random index date was assigned to each fracture-free individual matched with a patient in the fracture cohort, based on distribution of index date for cases. Individuals in the nonfracture cohort were also divided into 4 main categories (described above) based on the fracture site of a patient with a fracture to whom they were matched.

### Variables of interest and outcome measures

#### Cohort characteristics

Cohort characteristics included variables used for matching (described above), as well as osteoporosis, steroid, or opioid treatment 1 yr prior to index date and type of institution where fracture management occurred.

### Outcome measures

Deaths due to any cause were collected from the Registered Persons Database for both cohorts from the time of index date occurring between January 2011 and March 2015 until the end of study follow-up in March 2017. Thus, the length of follow-up for each participant depended on their index date. Minimum follow-up was 2 yr in all participants assessed in this study (ie, even those recruited in 2015 had at least 2 yr of follow-up), and maximum follow-up was 6 yr, which included a smaller number of participants with index date occurring in 2011 (approximately 20% of the cohort); the median length of follow-up was 3 yr.^3^

Additional outcomes relevant to the current analysis of postfracture survival, such as subsequent fracture(s) after index fracture,^20^ surgery rates (hip, 91%; vertebral, 9%; pNHNV, 6%–81%; dNHNV, 12%–28%),^3^ and other mortality outcomes,^3^ were reported in previous studies of this cohort. Compared to the previous report of mortality outcomes from this cohort, the current study excluded patients with multiple fractures (see Participants section above) and thus has a 3% (*n* = 3299) smaller study population than previously reported.^3^

### Statistical analysis

This was a descriptive study; thus, hypothesis testing and other formal comparisons using inferential statistics were not performed. Cohort characteristics and survival estimates were described separately for males and females and for the 4 index fracture-site categories described above (see Participants section). Three age subgroups (66–75, 76–85, and ≥ 86 yr) were additionally formed within each fracture-site category to characterize index fracture numbers and survival rates based on age. Kaplan–Meier estimates were used to conduct survival analysis. All-cause overall (ie, absolute) survival was obtained at 1 and 5 yr of follow-up after index date for fracture and nonfracture cohorts. To eliminate competing sources of deaths between the fracture and nonfracture cohorts and assess net postfracture survival,^17^ RSRs (fracture cohort overall survival/nonfracture cohort overall survival) were also obtained at 1 and 5 yr. Calculations for finding the derivative or integral of a curve were performed to empirically estimate the inflection point in the survival or Kaplan–Meier functions and determine when the greatest change in survival occurred.^25^ The inflection point was, in this case, the time at which the greatest change was observed while plotting a survival curve. Finally, Epanechnikov kernel–smoothed hazard function was plotted, based on time after the index date, to model which post-index date periods have the highest versus lowest chances of death.^26^ The hazard function is also known as instantaneous death rate or force of mortality because hazard is the probability of the event (eg, death) occurring during any given time point. Because CIs are based on smoothing of all postfracture data, large CIs can be observed at the end of follow-up, and thus Epanechnikov kernel–smoothed hazard function plots were provided with and without CIs.

## Results

### Fracture and nonfracture cohorts

**Figure 1:**
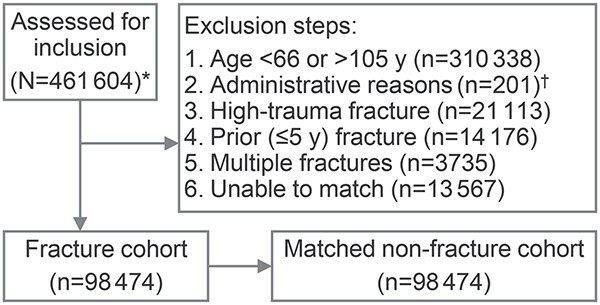
Flow of participants in the fracture and nonfracture cohorts. The fracture and nonfracture cohorts used in this study were previously described.^3^^,^^18-20^^*^Individuals assessed for inclusion in the fracture cohort sustained an index fracture between January 1, 2011, and March 31, 2015, at an osteoporotic fracture site (hip, clinical vertebral, pNHNV [pelvis, femur, sternum/rib/clavicle, and humerus/shoulder], and dNHNV [tibia/fibula/knee, radius/ulna, and wrist]) identified from hospital admissions, emergency room visits, and ambulatory care using International Classification of Diseases, 10th revision, Canada codes (Supplementary Methods). Individuals assessed for inclusion in the nonfracture cohort did not experience a fracture during and 5 yr before the index period and were matched to the fracture cohort on the following prespecified variables: sex, age group, rural/urban residence, and comorbidities associated with fracture risk. ^†^Death date prior to index fracture date (*n* = 78) or non-Ontario resident (*n* = 123). dNHNV, distal non-hip non-vertebral; pNHNV, proximal non-hip non-vertebral.

Of 461 604 individuals assessed for inclusion based on having experienced a fracture between 2011 and 2015 at a site associated with osteoporosis, 98 474 patients (73% women) were included in the fracture cohort and matched 1:1 to the nonfracture cohort (Figure 1). Fracture and nonfracture cohorts had the same number and distribution of individuals based on age groups, sex, and comorbidities (Supplementary Table 1).

### Patients with fracture

The median (interquartile range) age at index fracture was 80 (73–87) yr, with the lowest median age (75 yr) observed in males with a dNHNV fracture and the highest (85 yr) in females with a hip fracture (Table 1). When inspecting comorbidities typically associated with a high mortality, cancer (4%–5% females, 7%–9% males), myocardial infarction (3%–4% females, 6%–9% males), and CKD (in females only; 6%–9%) were each observed in <10% of the fracture (and nonfracture) cohort, whereas CKD in males (10%–15%), COPD (20%–28% females, 25%–35% males), stroke (20%–31% females, 24%–37% males), and diabetes (24%–28% females, 34%–36% males) were each observed at higher rates. Furthermore, COPD and stroke tended to be more common in the pNHNV, vertebral, and hip-fracture groups relative to the dNHNV fracture group, especially in males. During the year preceding an index fracture, 32% to 45% of female patients and 7% to 14% of male patients received osteoporosis treatment, with numerically higher treatment rates observed among patients with vertebral fracture. Vertebral fracture was also associated with the highest rates of opioid treatment history relative to the other fractures. Finally, fractures were primarily managed in this cohort in a large community or teaching hospital.

**Table 1:** Fracture cohort characteristics.

**Characteristic**	**Females**				**Males**			
	**Hip fracture**	**Vertebral fracture**	**pNHNV fracture**	**dNHNV fracture**	**Hip fracture**	**Vertebral fracture**	**pNHNV fracture**	**dNHNV fracture**
	*n* = 19 229	*n* = 4575	*n* = 23 636	*n* = 24 485	*n* = 7734	*n* = 2020	*n* = 10 577	*n* = 6218
Age, yr								
Median (IQR)	85 (79–90)	83 (76–88)	81 (74–87)	76 (70–83)	83 (77–88)	82 (75–87)	79 (72–85)	75 (70–82)
Mean (SD)	84.0 (7.67)	81.9 (7.69)	81.0 (8.29)	77.0 (7.97)	82.4 (7.72)	81.1 (7.82)	78.8 (8.01)	76.0 (7.39)
Respiratory conditions^a^								
Asthma	2442 (12.7)	749 (16.4)	3358 (14.2)	3492 (14.3)	692 (8.9)	224 (11.1)	1095 (10.4)	625 (10.1)
COPD	4898 (25.5)	1259 (27.5)	5830 (24.7)	4957 (20.2)	2549 (33.0)	709 (35.1)	3325 (31.4)	1564 (25.2)
Inflammatory conditions^a^								
RA	444 (2.3)	139 (3.0)	723 (3.1)	595 (2.4)	52 (0.7)	19 (0.9)	95 (0.9)	46 (0.7)
Psoriasis	865 (4.5)	240 (5.2)	1166 (4.9)	1273 (5.2)	290 (3.7)	91 (4.5)	560 (5.3)	332 (5.3)
SPA	470 (2.4)	151 (3.3)	583 (2.5)	561 (2.3)	140 (1.8)	66 (3.3)	252 (2.4)	140 (2.3)
Cancer^a^	825 (4.3)	238 (5.2)	1077 (4.6)	957 (3.9)	550 (7.1)	173 (8.6)	758 (7.2)	439 (7.1)
CKD^a^	1716 (8.9)	392 (8.6)	1922 (8.1)	1348 (5.5)	1164 (15.1)	241 (11.9)	1229 (11.6)	596 (9.6)
Diabetes^a^	5086 (26.4)	1265 (27.7)	6677 (28.2)	5790 (23.6)	2613 (33.8)	711 (35.2)	3848 (36.4)	2195 (35.3)
Vascular events^a^								
MI	790 (4.1)	175 (3.8)	843 (3.6)	634 (2.6)	594 (7.7)	177 (8.8)	820 (7.8)	390 (6.3)
Stroke	5920 (30.8)	1403 (30.7)	6432 (27.2)	4957 (20.2)	2891 (37.4)	709 (35.1)	3315 (31.3)	1473 (23.7)
Osteoarthritis^a^	15 668 (81.5)	3769 (82.4)	19 165 (81.1)	18 121 (74.0)	5570 (72.0)	1428 (70.7)	7238 (68.4)	3953 (63.6)
Dementia^a^	5709 (29.7)	765 (16.7)	4385 (18.6)	2631 (10.7)	1994 (25.8)	322 (15.9)	1429 (13.5)	494 (7.9)
Steroid treatment^b^	424 (2.2)	218 (4.8)	635 (2.7)	372 (1.5)	195 (2.5)	115 (5.7)	279 (2.6)	112 (1.8)
Opioid treatment^b^	5359 (27.9)	1995 (43.6)	7151 (30.3)	5800 (23.7)	2154 (27.9)	842 (41.7)	3168 (30.0)	1587 (25.5)
Osteoporosis treatment^b^	6086 (31.7)	2074 (45.3)	9114 (38.6)	8119 (33.2)	724 (9.4)	289 (14.3)	980 (9.3)	464 (7.5)
Fracture treatment institution								
Teaching	4868 (25.3)	1061 (23.2)	5592 (23.7)	5556 (22.7)	2023 (26.2)	532 (26.3)	2478 (23.4)	1480 (23.8)
Large community	13 584 (70.6)	3253 (71.1)	16 524 (69.9)	17 073 (69.7)	5420 (70.1)	1361 (67.4)	7291 (68.9)	4257 (68.5)
Small community	750 (3.9)	190 (4.2)	1212 (5.1)	1302 (5.3)	282 (3.6)	96 (4.8)	645 (6.1)	345 (5.5)
Missing	27 (0.1)	71 (1.6)	308 (1.3)	554 (2.3)	9 (0.1)	31 (1.5)	163 (1.5)	136 (2.2)

COPD, chronic obstructive pulmonary disease; dNHNV, distal non-hip non-vertebral; IQR, interquartile range; MI, myocardial infarction; pNHNV, proximal non-hip non-vertebral; RA, rheumatoid arthritis; SPA, spondyloarthritis.

Data are expressed as *n* (%) unless indicated otherwise.

a Any time before index date, except for cancer, which was ≤5 yr before index date.

b Within 1 yr before index date. Osteoporosis treatments included antiresorptive therapies or hormone replacement therapies (in 3%–5% of females); bone-formation agents were not publicly covered in Ontario during the index period.

**Figure 2:**
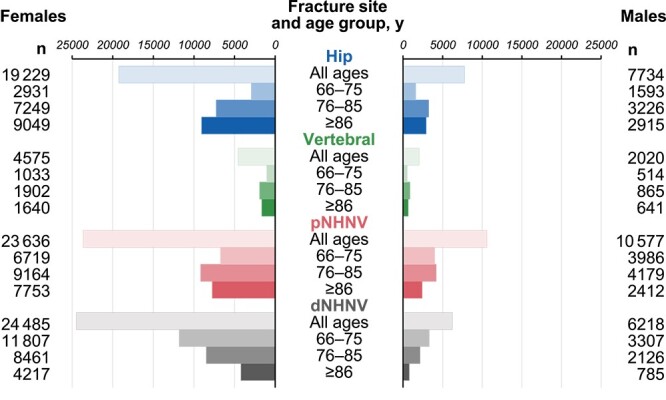
Number of females and males with index fracture by fracture site category and age group. dNHNV, distal non-hip non-vertebral; pNHNV, proximal non-hip non-vertebral.

As expected, more female patients than male patients experienced a fracture during the indexing period, with the difference being severalfold in magnitude and depending on fracture-site category and age (Figure 2). Overall, the most common fractures among all fracture cohort patients aged ≥66 yr were dNHNV and pNHNV fractures in females and pNHNV in males. However, this cohort exhibited the classic “fracture continuum,”^27^ especially in female patients, whereby the most common fractures were dNHNV fractures in the youngest age group, pNHNV fractures in the middle age groups, and hip fractures in the oldest age group. Vertebral fractures were identified based on clinical presentation and overall were the least common fractures, with relatively similar numbers occurring among the 3 age groups in both sexes.

### Post-fracture survival

#### Fracture site

**Figure 3:**
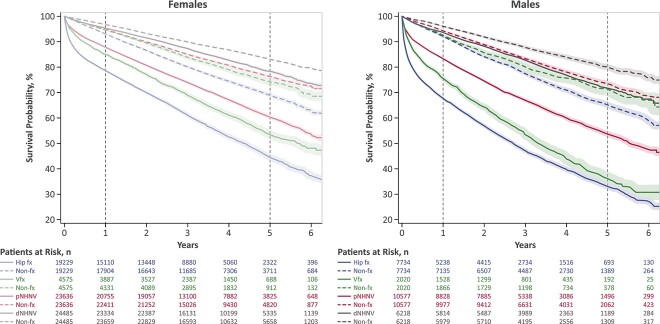
Survival probability in matched fracture and nonfracture cohorts. Dotted curves indicate nonfracture groups and are color-matched to their respective fracture cohort group. Number of patients at risk in nonfracture groups are labeled as “non-fx” and are listed directly below and color-matched to their respective fracture group. X-axis 0 indicates index date. Shaded regions represent 95% CIs. Dotted gray vertical lines indicate 1 and 5 yr of follow-up after index date. dNHNV, distal non-hip non-vertebral; fx, fracture; non-fx, nonfracture; pNHNV, proximal non-hip non-vertebral; Vfx, vertebral fracture.

Survival probability was the lowest after a hip fracture, followed by vertebral and pNHNV fractures, whereas patients with a dNHNV fracture showed similar survival probability as matched nonfracture individuals (Figure 3). Male patients tended to have lower survival probability than females after hip, vertebral, and pNHNV fractures, and the sex difference was particularly visible after a vertebral fracture, with males experiencing similar survival probability as after a hip fracture.

#### Time after fracture

**Figure 4:**
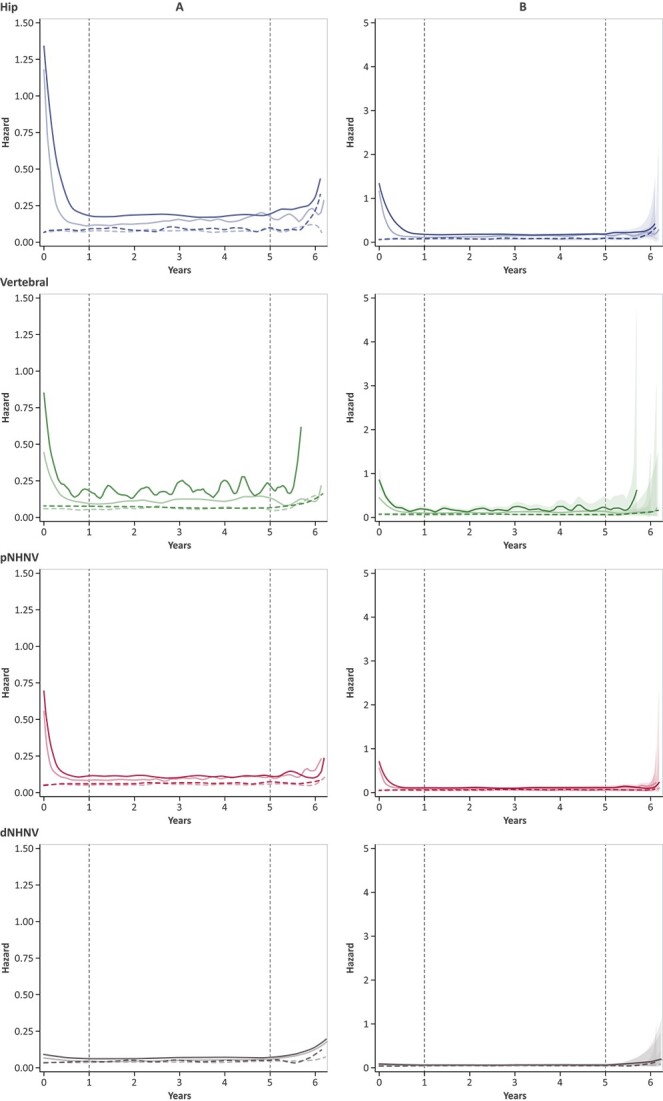
Epanechnikov kernel–smoothed hazard functions in matched fracture and nonfracture females and males without CIs* and y-axis of 1.50 (A) and with 95% CIs (shaded regions) and y-axis of 5 (B). Lighter color curves indicate females, darker color curves indicate males, dotted curves indicate nonfracture cohort. X-axis 0 indicates index date. Shaded regions represent 95% CIs. Dotted gray vertical lines indicate 1 and 5 yr of follow-up after index date. ^*^Based on Epanechnikov kernel smoothing of postfracture data over 6 yr of follow-up, large CIs were observed at the end of follow-up, and thus hazard function plots are provided without CIs in the A panel to support visual inspection of plots. dNHNV, distal non-hip non-vertebral; pNHNV, proximal non-hip non-vertebral.

The largest separation of survival curves occurred between fracture and nonfracture cohorts within the first year of follow-up after hip, vertebral, and pNHNV fracture, with the separation persisting over 5 yr of follow-up (Figure 3). The inflection point in the survival function, which was empirically estimated using the first derivative, demonstrated that the greatest reduction in survival occurred within 1 mo after hip, vertebral, and pNHNV fractures for both males and females. This timing was also confirmed by the second derivative by identifying the time at which the second derivative was equal to 0. Visual inspection of the hazard function also showed that the instantaneous probability of death was the highest immediately after a hip, vertebral, or pNHNV fracture (Figure 4A without CIs and small y-axis, Figure 4B with 95% CI and larger y-axis). After the immediate initial increase, the instantaneous probability of death declined by the end of the first postfracture year, returning to a level close above that of nonfracture individuals, and remaining relatively constant over 5 yr of follow-up for patients with hip and pNHNV fracture (both females and males), and females with vertebral fracture. In contrast, fluctuations in the risk of death were observed following the first year after vertebral fracture in males.

#### Overall survival

**Figure 5:**
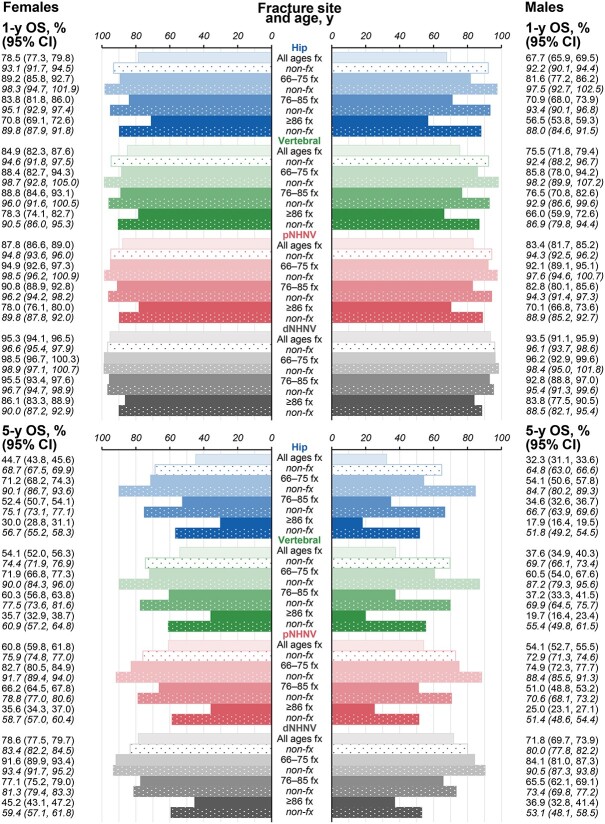
Overall survival at 1 and 5 yr in matched fracture and nonfracture females and males. Nonfracture cohort data and labels are in italic font positioned directly below their respective fracture group and in dotted bars color-matched to their respective fracture cohort group. dNHNV, distal non-hip non-vertebral; fx, fracture cohort; non-fx, nonfracture cohort; OS, overall survival; pNHNV, proximal non-hip non-vertebral.

Depending on fracture site and sex, overall survival ranged substantially from 67.7% to 95.3% at 1 yr and from 32.3% to 78.6% at 5 yr of follow-up among fracture cohort patients aged ≥66 yr (Figure 5). In the entire fracture cohort, less than a third of males and half of females survived 5 yr after a hip fracture, with overall survival rates being marginally better following vertebral fracture. The oldest patients (age ≥86 yr) had the worst prognosis, with the lowest 1- and 5-yr overall survival rates observed in this age group after hip fractures in males (56.5% and 17.9%), followed by vertebral fractures in males (66.0% and 19.7%), and hip fracture in females (70.8% and 30.0%). In contrast, overall survival remained above 90% and within a relatively narrow range (92.2%–96.6%) at 1 yr of follow-up in all nonfracture cohort individuals aged ≥66 yr, regardless of sex and fracture-site matching. At 5 yr of follow-up, overall survival ranged more widely in the nonfracture individuals aged ≥66 yr (64.8%–83.4%) and was the lowest among the oldest individuals matched to patients with a hip fracture. Although in the nonfracture cohort males tended to have lower overall survival at 5 yr of follow-up relative to females, the sex differences appeared to be smaller than in the fracture cohort.

#### Relative survival

**Figure 6:**
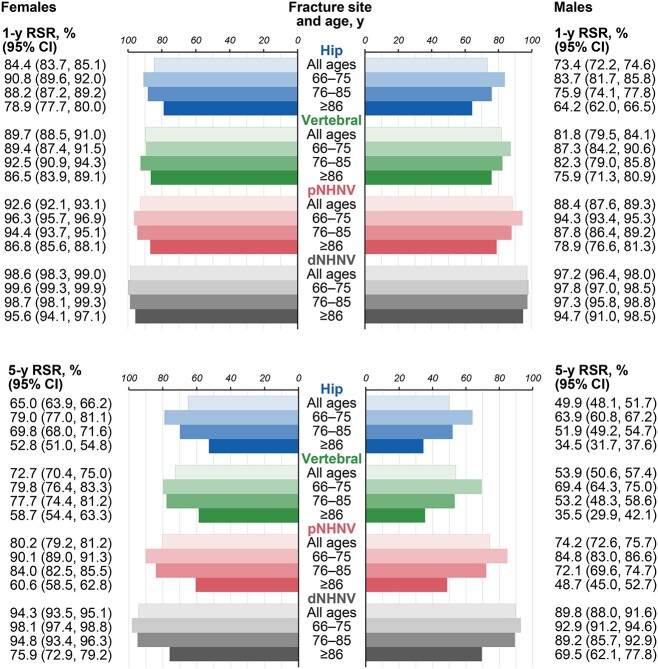
RSRs at 1 and 5 yr post-fracture in females and males. dNHNV, distal non-hip non-vertebral; RSR, relative survival ratio; pNHNV, proximal non-hip non-vertebral.

Relative survival analysis showed that, after accounting for competing sources of death in the fracture and nonfracture cohorts, the overall fracture survival trends remained, and 1- and 5-yr RSRs were observed to be lower when occurring at fracture sites associated with more severe fractures (ie, hip, clinical vertebral, and pNHNV), in males, and in older age groups (Figure 6). At 5 yr postfracture, the lowest RSRs were observed after hip fractures in males (age 66–85 yr, 51.9%–63.9%; age ≥86 yr, 34.5%), followed closely by vertebral fractures in males (age 66–85 yr, 53.2%–69.4%; age ≥86 yr, 35.5%), and then hip fractures in females (age 66–85 yr, 69.8%–79.0%; age ≥86 yr, 52.8%). Although RSRs did not decrease as markedly after dNHNV fractures in younger patients, relatively low 5-yr RSRs were observed in females (75.9%) and males (69.5%) aged ≥86 yr, emphasizing poor survival in this patient population regardless of sex and fracture-site category.

Additional observations made as part of the relative survival analysis included vertebral fractures having similar RSRs as hip fractures in certain subgroups; namely, older males (age ≥76 yr) at 5 yr of follow-up and the youngest females (age 66–75 yr) at 1 or 5 yr of follow-up. The youngest female patients with a vertebral fracture deviated slightly from the general sex/age trends observed in this study, with their 1-yr RSR (89.4%) being slightly worse than in older females (age 76–85 yr; 92.5%) and relatively similar to that of the youngest males (age 66–75 yr; 87.3%). Meanwhile, worse postfracture survival in male relative to female patients was particularly visible in the oldest males (age ≥86 yr) at 5 yr after a vertebral fracture (35.5% vs 58.7%, respectively).

## Discussion

In this real-world population-based study of 98 474 adults aged ≥66 yr, reduced RSRs were observed at 1 and 5 yr after a hip, clinical vertebral, and pNHNV fracture, to the extent that varied in relation to age, sex, and fracture location category. Postfracture RSRs have rarely been reported, especially in the last decade,^6-9^ with these results thus providing a comprehensive update on expected post-fracture prognosis. The greatest reduction in survival was found to occur within the first month after hip, vertebral, and pNHNV fractures for both males and females, which is in line with recent research^15^^,^^28^ and suggests a high relative impact of short-term factors on survival, with the effects persisting over time.^29^ Therefore, the most critical period for implementing clinical interventions aimed at improving post-fracture prognosis appears to be immediately after a fracture occurs, ideally before patients are discharged from fracture care. However, considering the immediate need for introducing secondary prevention strategies, primary fracture prevention is also crucial to prevent a fracture from occurring in the first place.

Excess postfracture mortality in adults aged ≥65 yr is understood to be multifactorial.^2^ A subsequent fracture is a potential short-term driver of reduced survival within the initial year owing to observations of imminent fracture risk^20^^,^^30-35^ and subsequent fracture being a predictor of mortality after an initial fracture.^1^^,^^12^^,^^36-38^ Perioperative complications in surgically treated patients have also been shown to increase mortality in the initial postfracture months^39-42^ and can potentially interact with other factors including severity of injury, duration of bedrest, comorbidities status, and old age. In the long term, frailty is associated with excess mortality^43-45^ and its progression can advance after a fracture,^44^^,^^46^ with some proposing that frailty and fractures potentially interact in a vicious cycle.^47^^,^^48^

Strategies for addressing the immediate decline in post-fracture survival may include preventing perioperative complications, some of which have already been found to be successful for patients with a hip fracture. Limiting time to surgery has been shown to provide mortality benefits for these patients,^42^ as well as the delivery of multidisciplinary orthogeriatric care that includes postoperative management of delirium and other conditions, nutritional counselling, and rehabilitation.^49-51^ However, these strategies have thus far focused on patients with hip fractures and not on other vulnerable fracture patient populations, while the multidisciplinary orthogeriatric care model has yet to become a standard of care in many regions. Immediate strategies should also consider pharmacologic treatments to reduce imminent fracture risk.^31^^,^^32^^,^^34^ Recently, a large population-based observational study has shown mortality benefits for various types of treatments^52^^,^^53^; albeit, not without methodological limitations inherent to observational study design^54^ and with adequately designed randomized trials examining all-cause mortality being scarce.^55^ An absence of prior antiosteoporosis treatment was, however, observed to be associated with increased postfracture mortality,^12^ and a clinical pathway focusing on improving post-fracture treatment rates, known as fracture liaison service, has also been shown to reduce mortality.^56^^,^^57^ However, fracture liaison service is typically implemented at institutions by self-selected hospital champions using finite external funding, because it is currently not a standard practice to assess and treat postfracture patients by the time they are discharged from fracture care. Current postfracture assessment and treatment rates thus remain alarmingly low.^11^^,^^20^^,^^50^ Considering that postfracture treatment rates are so dismal, public health agencies have currently prioritized secondary fracture prevention in adults aged ≥50 yr to more efficiently deliver resources and focus on a smaller number of patients who have the highest subsequent fracture risk due to a recent fracture.^11^ Nevertheless, this prioritization should not come at the expense of reducing focus on primary fracture prevention to minimize the risk of initial fracture in adults aged ≥50 yr, through screening for osteoporosis and fracture-risk factors with simple tools such as the FRAX calculator.^11^^,^^31^ In terms of secondary fracture prevention, all patients aged ≥50 yr with a fracture experienced at a site typically associated with osteoporosis should be assessed for fracture risk and considered for appropriate treatment depending on their risk level.^31^^,^^32^^,^^34^ Importantly, even patients with a dNHNV fracture should be assessed, since these fractures increase the risk of subsequent fracture(s),^31^ as well as patients with a fracture occurring due to high trauma, since high-trauma fractures were recently also observed to increase subsequent fracture risk in older adults.^24^

However, certain patient populations were found to have a relatively poor post-fracture survival and may need to be prioritized with higher urgency. First, patients with more severe fractures were found to have a poor survival rate, confirming results of previous studies that observed especially poor mortality outcomes after hip, vertebral, and certain pNHNV fractures, including femur, pelvis, humerus, and multiple rib fractures.^12-15^ This is not surprising considering that more severe fractures tend to involve larger bones and/or are located more proximally to the body’s core organs and tissues and thus are associated with more serious injury. More severe fracture locations may also lead to prolonged mobility issues and/or bed rest, which may also worsen longer term prognosis. Hip fractures were associated with a particularly poor survival rate, especially in the oldest patients, who also tend to have a high risk of experiencing a hip fracture. In many countries, hip fracture numbers are expected to rise with the rapidly aging population,^58^ further highlighting the need for secondary fracture prevention initiated at discharge. A large portion of hip-fracture patients have a history of fracture^59^ that can be used as a signal to intervene and help reduce subsequent hip-fracture risk.

Another patient group associated with a relatively poor postfracture prognosis included male patients, who are postulated to be sicker or frailer than female patients prior to their fracture (due to having generally lower fracture risk)^2^^,^^3^ and who also tend to have significantly lower osteoporosis treatment rates relative to females.^20^ This sex difference was especially visible in this study after a hip or vertebral fracture. Men aged 66 to 85 and ≥86 yr, respectively, were observed to have a 5-yr RSR ranging from 52% to 69% and 35% to 36% after a hip or vertebral fracture, compared to 70% to 80% and 53% to 59% among their female counterparts. To put these postfracture RSRs in context of other burdensome diseases in older males and females, these data were inspected relative to another cohort of Ontario residents assessed after a cancer diagnosis from 2012 to 2016.^16^ The following 5-yr RSRs were reported after a diagnosis of different types of cancer relevant to older adults: 64% in adults aged 60 to 79 yr with any type of cancer, 43% in adults aged ≥80 yr with any type of cancer, 94% in males (any age) with prostate cancer, and 89% in females (any age) with breast cancer.^16^ These between-study comparisons indicate that postfracture prognosis is similar to that observed after common types of cancer in older men, which also aligns with previous within-study comparisons observing that the proportion of deaths that could be attributed to fracture in men aged ≥45 yr was similar to that attributed to cancer (and less than cardiovascular disease).^5^ However, it is crucial to note that postfracture mortality outcomes in women were previously reported to resemble their cardiovascular disease mortality outcomes,^3^ with the aforementioned study observing that although the proportion of deaths attributed to fracture in women was lower than that attributed to cancer, it resembled that of cardiovascular disease.^5^ Furthermore, although female patients tend to have better postfracture survival than males, women have a significantly higher fracture risk. Thus, in this cohort, female fracture patients, who exhibited the classic “fracture continuum,”^27^ contributed to two-thirds of postfracture deaths.^3^

A final patient group observed to have particularly poor postfracture prognoses were the oldest patients (≥86 yr), who had reduced RSRs even after dNHNV fractures. This is an important observation, because an erroneous impression often persists among patients, caregivers, and clinicians that older adults can just as easily recover after a dNHNV fracture as younger patients. Interestingly, this observation of worse post-fracture survival in older age groups contradicts prior studies reporting higher relative mortality risk in younger versus older populations.^2^^,^^3^ These seemingly discrepant findings highlight the need to report survival rates along with other mortality outcomes, as commonly done in other disease areas, since different mortality statistics convey slightly different information.^16^^,^^17^ A higher relative mortality risk in younger versus older fracture patients can be explained by low mortality risk typically found in younger patients relative to older individuals without a fracture, which thereby inflates the relative mortality risk for younger individuals with a fracture.^2^^,^^3^ However, worse postfracture survival in older relative to younger patients is not surprising, given that older patients are generally more vulnerable to and less resilient in recovery from substantial physical stresses, such as fractures.

The strengths of this study include having a relatively high generalizability to the population of Ontario, since the source data encompass publicly funded health services records for the population of Ontario, with its residents being eligible for universal public health coverage since 1986.^23^ Ontario is the most populous Canadian province (>15 million) and, like many areas around the world, has a rapidly aging population.^60^^,^^61^ Adults aged ≥65 yr are currently estimated to make up 18.5% of Ontario’s population, with their numbers projected to double by 2046 and make up approximately a quarter of the population.^61^^,^^62^ Ontario is also an ethnically diverse province, with approximately one-third of its population being a visible minority and 88% identifying as having a non-Canadian ethnic or cultural origin.^62^ Various mortality outcomes were reported in this cohort, including survival rates here and relative mortality risks in a previous study,^3^ thus providing robust mortality outcome data from the same population. Twelve comorbidities that commonly occur in older adults with increased fracture risk were used for matching and RSR calculations to better account for competing causes of death of fracture individuals versus nonfracture individuals. The exclusion of patients with high-trauma fractures helped focus on low-trauma or fragility fractures in this study and minimized potential confounding effects of soft-tissue injury and systemic effects on survival due to high trauma; albeit, recent evidence shows that not only fragility fractures but also high-trauma fractures are associated with subsequent fracture risk in older adults and should prompt clinicians to perform fracture-risk assessment.^24^

Limitations of our study include those inherent to a descriptive study and to the use of administrative databases and coding. As with any study relying on administrative data, unmeasured confounders may still be present after matching. ICD codes identified patients with fractures, and as such, only symptomatic vertebral fractures were included. However, approximately two-thirds of vertebral fractures are estimated to be silent, and opportunistically identified silent vertebral fractures have also been found to increase mortality.^63^^,^^64^ Therefore, a relatively small number of patients with clinical vertebral fracture were present in this study, especially during the 5th or 6th year of follow-up. Patients who experienced multiple fractures during the same incident were excluded from this analysis, as they could not be categorized in 1 of the 4 fracture-site categories based on a single fracture site; however, because these patients were also found to have a relatively high 1-yr mortality risk in a previous analysis of this cohort,^3^ future studies of post-fracture mortality outcomes should examine these patients as a stand-alone group. Racial and ethnic data were not collected for inclusion in baseline characteristics owing to unavailable data, and thus, findings may not be generalizable to specific races or ethnicities. Cause-of-death information was not available; therefore, all-cause deaths were assessed in this study and may not be fully attributable to the index fracture event. Therefore, stratification by cause of death may be an important future research direction. Additional stratifications that may be important to examine in future research, to identify patients with poor prognosis for each fracture site, include orthopedic management (ie, conservative vs surgical), comorbidities status (eg, Charlson Comorbidity Index), and frailty status.

In conclusion, this study demonstrates the need for reporting survival rates in older adults, in addition to other postfracture mortality outcomes, to help monitor improvements in postfracture prognosis over time. The most critical period for implementing clinical interventions aimed at improving postfracture prognosis appeared to be immediately after a fracture, ideally within the first postfracture month, before patients are discharged from fracture care. Targeting patients with a fracture by the time they are discharged from fracture care can potentially utilize similar clinical pathways as those used in the cardiovascular setting, whereby specialists are engaged within a hospital for assessment prior to patient discharge. Unlike cardiovascular disease management, such clinical pathways do not currently exist as a standard at most hospitals.

## Supplementary Material

Supplementary_Material_ziae002

## Data Availability

The datasets generated during and/or analyzed during the current study are available in the ICES repository upon request. The data that support the findings of this study are available from ICES. However, restrictions apply to the availability of these data, which were used under license for the current study and therefore are not publicly available [https://www.ices.on.ca/data-privacy/]. Data are, however, available from the authors upon reasonable request and with permission from ICES.
