# Supplementary material for: Postfracture survival in a population-based study of adults aged ≥66 yr: a call to action at hospital discharge
Source: JBMR Plus. 2024 Apr 9;8(5):ziae002. doi: 10.1093/jbmrpl/ziae002 (PMC11001756; doi:10.1093/jbmrpl/ziae002)
Supplement: Supplementary_Material_ziae002 [file supplementary_material_ziae002.docx]

# Supplementary Appendix

# Supplementary Methods

## International Classification of Diseases, 10th Revision, Canada (ICD-10-CA) Codes Used for Identification of Index Fractures and Their Categories Based on Fracture Site

| **Fracture Type** | **ICD-10-CA Code** |
| --- | --- |
| **INCLUDED FRACTURE CODES** | |
| **Hip Fracture** | |
| Hip | S72.0 - Fracture of neck of femur  S72.1 - Pertrochanteric fracture   - Intertrochanteric fracture - Trochanteric fracture   S72.2 - Subtrochanteric fracture |
| **Vertebral Fracture** | |
| Vertebral | S22.0 - Fracture of thoracic vertebra, fracture of thoracic spine NOS  S22.1 - Multiple fractures of thoracic spine  S32.0 - Fracture of lumbar vertebra, fracture of lumbar spine |
| **Proximal Non-hip Non-vertebral (pNHNV) Fracture** | |
| Sternum, ribs, and clavicle | S22.2 - Fracture of sternum  S42.0 - Fracture of clavicle  S22.3 - Fracture of rib  S22.4 - Multiple fractures of rib |
| Pelvis | S32.1 - Fracture of sacrum  S32.2 - Fracture of coccyx  S32.3 - Fracture of ilium  S32.4 - Fracture of acetabulum  S32.5 - Fracture of pubis  S32.7 - Multiple fractures of lumbar spine and pelvis  S32.8 - Fracture of other and unspecified parts of lumbar spine and pelvis   - Fracture of   - Ischium   - Lumbosacral spine NOS   - Pelvis NOS |
| Femur | S72.3 - Fracture of shaft of femur  S72.4 - Fracture of distal end of femur  S72.7 - Multiple fractures of femur  S72.8 - Fractures of other parts of femur  S72.9 - Fracture of femur, part unspecified   - Applicable to fracture of thigh NOS Fracture of upper leg NOS - Type 1 exclusion for fracture of hip NOS |
| Humerus/ shoulder | S42.2 - Fracture of upper end of humerus  S42.3 - Fracture of shaft of humerus  S42.4 - Fracture of lower end of humerus  S42.8 - Fracture of other parts of shoulder and upper arm  S42.9 - Fracture of shoulder girdle, part unspecified  Fracture of shoulder NOS |
| **Distal Non-hip Non-vertebral (dNHNV) Fracture** | |
| Radius and ulna | S52.0 - Fracture of upper end of ulna  S52.1 - Fracture of upper end of radius  S52.2 - Fracture of shaft of ulna  S52.3 - Fracture of shaft of radius  S52.4 - Fracture of shafts of both ulna and radius  S52.7 - Multiple fractures of forearm  S52.8 - Fracture of other parts of forearm   - Lower end of ulna - Head of ulna   S52.9 - Fracture of forearm, part unspecified |
| Wrist | S52.5 - Fracture of lower end of radius  S52.6 - Fracture of lower end of both ulna and radius |
| Tibia, fibula, and knee | S82.0 - Fracture of patella   - Kneecap   S82.1 - Fracture of upper end of tibia   - With or without mention of fracture of fibula   S82.2 - Fracture of shaft of tibia   - With or without mention of fracture of fibula   S82.3 - Fracture of lower end of tibia   - With or without mention of fracture of fibula   S82.4 - Fracture of fibula alone  S82.5 - Fracture of medial malleolus   - Tibia involving   S82.6 - Fracture of lateral malleolus   - Fibula involving |
| **Excluded Fracture Codes** | |
| Multiple fracture | Combination of fracture codes  S42.7 - Multiple fractures of clavicle, scapula, and humerus  T02.1 - Fractures involving thorax with lower back and pelvis  T02.2 - Fractures involving multiple regions of one upper limb  T02.3 - Fractures involving multiple regions of one lower limb  T02.4 - Fractures involving multiple regions of both upper limbs  T02.5 - Fractures involving multiple regions of both lower limbs  T02.6 - Fractures involving multiple regions of upper limb(s) with lower limb(s)  T02.7 - Fractures involving thorax with lower back and pelvis with limb(s)  T02.8 - Fractures involving other combinations of body regions  T02.9 - Multiple fractures, unspecified |

NOS, not otherwise specified.

Supplementary Table 1. Fracture Cohort and Nonfracture Cohort Matching for Each Fracture Site

| **Characteristic, n (%)** | **Hip  Fracture** | **No Hip  Fracture** | **Vertebral Fracture** | **No Vertebral Fracture** | **pNHNV Fracture** | **No pNHNV Fracture** | **dNHNV Fracture** | **No dNHNV Fracture** |
| --- | --- | --- | --- | --- | --- | --- | --- | --- |
| **FEMALES** | **n=19 229** | **n=19 229** | **n=4575** | **n=4575** | **n=23 636** | **n=23 636** | **n=24 485** | **n=24 485** |
| Age groups, y |  |  |  |  |  |  |  |  |
| *66–70* | 1224 (6.4) | 1224 (6.4) | 427 (9.3) | 427 (9.3) | 3330 (14.1) | 3330 (14.1) | 6493 (26.5) | 6493 (26.5) |
| *71–75* | 1707 (8.9) | 1707 (8.9) | 606 (13.2) | 606 (13.2) | 3389 (14.3) | 3389 (14.3) | 5314 (21.7) | 5314 (21.7) |
| *76–80* | 2848 (14.8) | 2848 (14.8) | 844 (18.4) | 844 (18.4) | 4215 (17.8) | 4215 (17.8) | 4534 (18.5) | 4534 (18.5) |
| *81–85* | 4401 (22.9) | 4401 (22.9) | 1058 (23.1) | 1058 (23.1) | 4949 (20.9) | 4949 (20.9) | 3927 (16.0) | 3927 (16.0) |
| ≥*86* | 9049 (47.1) | 9049 (47.1) | 1640 (35.8) | 1640 (35.8) | 7753 (32.8) | 7753 (32.8) | 4217 (17.2) | 4217 (17.2) |
| Respiratory conditions* |  |  |  |  |  |  |  |  |
| *Asthma* | 2442 (12.7) | 2442 (12.7) | 749 (16.4) | 749 (16.4) | 3358 (14.2) | 3358 (14.2) | 3492 (14.3) | 3492 (14.3) |
| *COPD* | 4898 (25.5) | 4898 (25.5) | 1259 (27.5) | 1259 (27.5) | 5830 (24.7) | 5830 (24.7) | 4957 (20.2) | 4957 (20.2) |
| Inflammatory conditions* |  |  |  |  |  |  |  |  |
| *RA* | 444 (2.3) | 444 (2.3) | 139 (3.0) | 139 (3.0) | 723 (3.1) | 723 (3.1) | 595 (2.4) | 595 (2.4) |
| *Psoriasis* | 865 (4.5) | 865 (4.5) | 240 (5.2) | 240 (5.2) | 1166 (4.9) | 1166 (4.9) | 1273 (5.2) | 1273 (5.2) |
| *SPA* | 470 (2.4) | 470 (2.4) | 151 (3.3) | 151 (3.3) | 583 (2.5) | 583 (2.5) | 561 (2.3) | 561 (2.3) |
| Cancer* | 825 (4.3) | 825 (4.3) | 238 (5.2) | 238 (5.2) | 1077 (4.6) | 1077 (4.6) | 957 (3.9) | 957 (3.9) |
| CKD* | 1716 (8.9) | 1716 (8.9) | 392 (8.6) | 392 (8.6) | 1922 (8.1) | 1922 (8.1) | 1348 (5.5) | 1348 (5.5) |
| Diabetes* | 5086 (26.4) | 5086 (26.4) | 1265 (27.7) | 1265 (27.7) | 6677 (28.2) | 6677 (28.2) | 5790 (23.6) | 5790 (23.6) |
| Vascular events* |  |  |  |  |  |  |  |  |
| *MI* | 790 (4.1) | 790 (4.1) | 175 (3.8) | 175 (3.8) | 843 (3.6) | 843 (3.6) | 634 (2.6) | 634 (2.6) |
| *Stroke* | 5920 (30.8) | 5920 (30.8) | 1403 (30.7) | 1403 (30.7) | 6432 (27.2) | 6432 (27.2) | 4957 (20.2) | 4957 (20.2) |
| Osteoarthritis* | 15 668 (81.5) | 15 668 (81.5) | 3769 (82.4) | 3769 (82.4) | 19 165 (81.1) | 19 165 (81.1) | 18 121 (74.0) | 18 121 (74.0) |
| Dementia* | 5709 (29.7) | 5709 (29.7) | 765 (16.7) | 765 (16.7) | 4385 (18.6) | 4385 (18.6) | 2631 (10.7) | 2631 (10.7) |
| **MALES** | **n=7734** | **n=7734** | **n=2020** | **n=2020** | **n=10 577** | **n=10 577** | **n=6218** | **n=6218** |
| Age groups, y |  |  |  |  |  |  |  |  |
| *66–70* | 689 (8.9) | 689 (8.9) | 254 (12.6) | 254 (12.6) | 2081 (19.7) | 2081 (19.7) | 1808 (29.1) | 1808 (29.1) |
| *71–75* | 904 (11.7) | 904 (11.7) | 260 (12.9) | 260 (12.9) | 1905 (18.0) | 1905 (18.0) | 1499 (24.1) | 1499 (24.1) |
| *76–80* | 1329 (17.2) | 1329 (17.2) | 391 (19.4) | 391 (19.4) | 2051 (19.4) | 2051 (19.4) | 1163 (18.7) | 1163 (18.7) |
| *81–85* | 1897 (24.5) | 1897 (24.5) | 474 (23.5) | 474 (23.5) | 2128 (20.1) | 2128 (20.1) | 963 (15.5) | 963 (15.5) |
| ≥*86* | 2915 (37.7) | 2915 (37.7) | 641 (31.7) | 641 (31.7) | 2412 (22.8) | 2412 (22.8) | 785 (12.6) | 785 (12.6) |
| Respiratory conditions* |  |  |  |  |  |  |  |  |
| *Asthma* | 692 (8.9) | 692 (8.9) | 224 (11.1) | 224 (11.1) | 1095 (10.4) | 1095 (10.4) | 625 (10.1) | 625 (10.1) |
| *COPD* | 2549 (33.0) | 2549 (33.0) | 709 (35.1) | 709 (35.1) | 3325 (31.4) | 3325 (31.4) | 1564 (25.2) | 1564 (25.2) |
| Inflammatory conditions* |  |  |  |  |  |  |  |  |
| *RA* | 52 (0.7) | 52 (0.7) | 19 (0.9) | 19 (0.9) | 95 (0.9) | 95 (0.9) | 46 (0.7) | 46 (0.7) |
| *Psoriasis* | 290 (3.7) | 290 (3.7) | 91 (4.5) | 91 (4.5) | 560 (5.3) | 560 (5.3) | 332 (5.3) | 332 (5.3) |
| *SPA* | 140 (1.8) | 140 (1.8) | 66 (3.3) | 66 (3.3) | 252 (2.4) | 252 (2.4) | 140 (2.3) | 140 (2.3) |
| Cancer* | 550 (7.1) | 550 (7.1) | 173 (8.6) | 173 (8.6) | 758 (7.2) | 758 (7.2) | 439 (7.1) | 439 (7.1) |
| CKD* | 1164 (15.1) | 1164 (15.1) | 241 (11.9) | 241 (11.9) | 1229 (11.6) | 1229 (11.6) | 596 (9.6) | 596 (9.6) |
| Diabetes* | 2613 (33.8) | 2613 (33.8) | 711 (35.2) | 711 (35.2) | 3848 (36.4) | 3848 (36.4) | 2195 (35.3) | 2195 (35.3) |
| Vascular events* |  |  |  |  |  |  |  |  |
| *MI* | 594 (7.7) | 594 (7.7) | 177 (8.8) | 177 (8.8) | 820 (7.8) | 820 (7.8) | 390 (6.3) | 390 (6.3) |
| *Stroke* | 2891 (37.4) | 2891 (37.4) | 709 (35.1) | 709 (35.1) | 3315 (31.3) | 3315 (31.3) | 1473 (23.7) | 1473 (23.7) |
| Osteoarthritis* | 5570 (72.0) | 5570 (72.0) | 1428 (70.7) | 1428 (70.7) | 7238 (68.4) | 7238 (68.4) | 3953 (63.6) | 3953 (63.6) |
| Dementia* | 1994 (25.8) | 1994 (25.8) | 322 (15.9) | 322 (15.9) | 1429 (13.5) | 1429 (13.5) | 494 (7.9) | 494 (7.9) |

CKD, chronic kidney disease; COPD, chronic obstructive pulmonary disease; dNHNV, distal nonhip nonvertebral; MI, myocardial infarction; pNHNV, proximal nonhip nonvertebral; RA, rheumatoid arthritis; SPA, spondyloarthritis.

*Any time before index date, except for cancer which was ≤5 years before index date.
